# Attitudes toward posthumous organ donation in Kazakhstan: a qualitative analysis

**DOI:** 10.3389/fpubh.2025.1602268

**Published:** 2025-06-30

**Authors:** Vitaliy Sazonov, Aruzhan Asanova, Aidos Bolatov, Saule Shaisultanova, Aigerim Abdiorazova, Yuriy Pya

**Affiliations:** ^1^“University Medical Center” Corporate Fund, Astana, Kazakhstan; ^2^Department of Surgery, School of Medicine, Nazarbayev University, Astana, Kazakhstan; ^3^Medical School, Shenzhen University, Shenzhen, China

**Keywords:** organ donation, public attitudes, Kazakhstan, cultural beliefs, transplantation, public health education

## Abstract

**Introduction:**

The present study aims to examine the attitudes of the Kazakhstani population toward posthumous organ donation using a mixed-methods approach.

**Methods:**

A cross-sectional survey of 1,345 participants representing diverse demographic backgrounds was conducted alongside a qualitative thematic analysis of open-ended responses to explore underlying motivations and barriers. Quantitative results indicate that more than one-third of respondents expressed willingness to donate, while approximately one-fifth declined, and the remaining participants preferred to leave the decision to their loved ones. Multinomial logistic regression revealed that factors such as increasing age, lower education level, higher religiosity, and language preference significantly influenced attitudes toward donation.

**Results and discussion:**

Qualitative analysis identified recurring themes, including religious and cultural concerns about the afterlife, uncertainty in decision making, ethical opposition rooted in tradition, distrust of the health care system, and the role of personal altruism. These findings underscore the complex interplay of socio-cultural and systemic factors that shape public perceptions of organ donation in Kazakhstan. Key barriers, such as religious concerns and mistrust of health care and cultural opposition, are identified, and potential solutions through education, policy change, and media engagement are outlined.

## 1 Introduction

Organ transplantation remains one of the most important advances in modern medicine, providing life-saving care to patients with end-stage organ failure (1). The availability of organs for transplantation depends on the registration and willingness of donors, making public attitudes toward organ donation a critical determinant of the success of transplantation programs. In many countries, increasing organ donation rates is an ongoing challenge, requiring policies and public health campaigns to increase awareness and acceptance of post-mortem donation (2).

However, the success of transplantation programs largely depends on organ donation rates, which are influenced by public attitudes, cultural norms, and religious beliefs (3). In Kazakhstan, organ donation remains a complex and often controversial issue, shaped by historical, religious and ethical considerations (4, 5).

Public reluctance to donate organs is often attributed to a combination of medical mistrust, limited knowledge, and deeply rooted cultural and spiritual perspectives. In addition, organ donation policies and legislation in Kazakhstan have undergone several reforms, which may have contributed to public uncertainty and reluctance to register as donors (6).

Despite medical advances and increasing awareness campaigns, the rate of organ donation in Kazakhstan remains relatively low. Existing studies suggest that socio-cultural factors, religious beliefs, and personal attitudes play a significant role in shaping public perceptions of posthumous organ donation. The presence of religious influence, particularly within Islamic communities, raises ethical concerns about the permissibility of organ donation and transplantation, further complicating public decision-making. In addition, misconceptions about the organ procurement process and fears of medical exploitation discourage individuals from becoming involved in organ donation (7).

However, comprehensive data on the specific barriers and motivators that influence organ donation decisions in Kazakhstan remain scarce (8). A deeper understanding of these factors is essential for the development of culturally sensitive strategies to increase public confidence and willingness to donate organs. It is crucial to examine not only the prevalence of positive and negative attitudes toward donation, but also the underlying reasons that drive these perspectives.

The present study aims to fill this gap by examining the attitudes of the Kazakhstani population toward posthumous organ donation using a mixed-methods approach. By integrating both quantitative survey data and qualitative thematic analysis, this study seeks to explore the underlying factors influencing public perspectives, identify key barriers and facilitators, and provide evidence-based recommendations for improving organ donation policies and awareness initiatives in Kazakhstan. The results of this study will contribute to a better understanding of attitudes towards organ donation and inform future efforts to increase donor registration rates and public support for transplantation programs. The knowledge gained from this research can help policymakers, healthcare professionals, and advocacy groups develop targeted interventions that are consistent with cultural and religious values while promoting the life-saving potential of organ transplantation.

## 2 Materials and methods

### 2.1 Study design

This study used a mixed-methods approach, integrating both quantitative and qualitative data collection techniques to assess attitudes toward post-mortem organ do-nation in Kazakhstan. A cross-sectional survey was conducted to capture a broad representation of public perspectives, supplemented by thematic analysis of open-ended responses to provide deeper insights into decision-making rationales.

Kazakhstan employs a lifetime opt-in consent system for post-humous organ donation. Citizens have the option to either consent to organ donation or explicitly prohibit it during their lifetime. However, even in cases where an individual has signed electronically an organ donation consent form, the final decision rests with the deceased’s relatives. This means that family members have the legal right to override the documented wishes of the deceased, potentially limiting the number of viable organ donations. This study explored public attitudes toward both personal decision making and the role of family influence in donation.

Respondents were asked about their personal decisions regarding posthumous organ donation and their reasons for either consenting or declining. An open-ended question–“Why did you make this decision?”–was included to allow participants to elaborate on their motivations, allowing for a deeper qualitative analysis of the underlying factors influencing donation decisions.

### 2.2 Participants and sampling

Participants were recruited from diverse demographic backgrounds, including urban and rural populations, healthcare professionals, and individuals with different religious affiliations. A non-probability convenience sampling method was used to maximize participation and ensure representation across age groups, education levels, and socioeconomic status. Eligibility criteria included being 18 years of age or older and residing in Kazakhstan at the time of the survey.

The survey consisted of both closed and open-ended questions. Demographic variables included age, gender, ethnicity, education level, religious affiliation, financial status, and occupational background. The open-ended responses were designed to explore personal motivations and concerns related to organ donation.

### 2.3 Data collection and ethical considerations

Data were collected via a self-administered online questionnaire distributed through social media platforms, university mailing lists, and professional and community networks using a convenience sampling strategy. Participation was voluntary, and informed consent was obtained from all respondents prior to completion of the survey. Ethical approval for the study was granted by the Local Bioethics Commission of the “University Medical Center” Corporate Fund (Protocol No. 3 dated July 14, 2023), ensuring compliance with ethical guidelines for human subjects research.

### 2.4 Data analysis

All statistical analyses were performed using R (version 4.4.2). Quantitative data were analyzed using descriptive statistics, including means, standard deviations, and absolute and relative frequencies. To compare participant characteristics by attitudes toward post-mortem organ donation, independent analysis of variance tests and chi-square tests were used. Multinomial logistic regression was applied to explore participant characteristics associated with attitudes toward organ donation. We included all participant characteristics in the model except for child presence and religion, due to their multicollinearity with the marital status and degree of religiosity variables, respectively. The model was tested for the linearity assumption by including transformed versions of the continuous age variable (e.g., squared, cubic), with no significant improvement in model performance. Collinearity was assessed by calculating the variance inflation factor (VIF). Similarly, no collinear variables (VIF < 4) were detected in the model. Thematic analysis of qualitative responses was conducted using an inductive coding approach to identify recurring themes and sentiment patterns. Four coders analyzed the responses independently. Any discrepancies were resolved through discussion. Although member checking was not feasible, triangulation and reflexive discussion enhanced analytical rigor. Triangulation of qualitative findings was conducted to increase the validity of the study’s conclusions. The qualitative research team consisted of male and female researchers with backgrounds in public health and medical ethics. They all had formal training in qualitative methods and had no prior relationships with the participants. Reflexive memos were kept to mitigate bias.

### 2.5 Reliability and validity

To ensure reliability, the survey instrument was piloted with a small sample prior to full implementation (9). The internal consistency of the attitudinal measures was assessed using Cronbach’s alpha. For validity, expert reviews were conducted to confirm the appropriateness of survey items in the cultural and ethical context of Kazakhstan.

## 3 Results

### 3.1 Sample characteristics

The final analytic sample comprised 1,345 fully completed questionnaires, with an average age of 37 ± 12 years (Table 1). Most respondents were female (78%), Kazakh (82%), predominantly spoke Kazakh (51%) or Russian (49%), had at least a bachelor’s degree (74%), were married (59%), and lived in urban areas (73%). The majority (72.2%) identified as Muslim, with an average religiosity score of 2.7 (where 1 represents not religious and 5 represents most religious). Over two-thirds of the respondents were healthcare providers.

**Table 1:** Characteristics of 1,345 survey participants by attitudes toward post-mortem organ donation in Kazakhstan.

Variable	Overall, *n* = 1,345	Agree to donate, *n* = 470	Leave the decision up to close relatives, *n* = 586	Refuse to donate, *n* = 289	*p*-value
Age, mean (SD)	36.7 (11.5)	32.6 (9.3)	38.4 (12.0)	40.2 (11.7)	<0.001
Sex* = Male, *n* (%)	291 (21.8)	96 (20.4)	121 (20.9)	74 (26.0)	0.162
Ethnicity, *n* (%)					0.353
Kazakh	1,096 (81.5)	391 (83.2)	481 (82.1)	224 (77.5)	
Russian	119 (8.8)	40 (8.5)	49 (8.4)	30 (10.4)	
Other	130 (9.7)	39 (8.3)	56 (9.6)	35 (12.1)	
Spoken language, *n* (%)					<0.001
Kazakh language	680 (50.6)	156 (33.2)	343 (58.5)	181 (62.6)	
Russian language	623 (46.3)	292 (62.1)	226 (38.6)	105 (36.3)	
Both languages or other language	42 (3.1)	22 (4.7)	17 (2.9)	3 (1.0)	
Education, *n* (%)					<0.001
High school or less	108 (8.0)	27 (5.7)	52 (8.9)	29 (10.0)	
Vocational	245 (18.2)	39 (8.3)	131 (22.4)	75 (26.0)	
Bachelor’s or Master’s	879 (65.4)	354 (75.3)	364 (62.1)	161 (55.7)	
Doctoral	113 (8.4)	50 (10.6)	39 (6.7)	24 (8.3)	
Residence = Urban, *n* (%)	1,066 (79.3)	408 (86.8)	437 (74.6)	221 (76.5)	<0.001
Marital status, *n* (%)					<0.001
Single	390 (29.0)	196 (41.7)	136 (23.2)	58 (20.1)	
Married	796 (59.2)	224 (47.7)	378 (64.5)	194 (67.1)	
Divorced	121 (9.0)	42 (8.9)	53 (9.0)	26 (9.0)	
Widowed	38 (2.8)	8 (1.7)	19 (3.2)	11 (3.8)	
Child presence = Yes, *n* (%)	891 (66.2)	224 (47.7)	440 (75.1)	227 (78.5)	<0.001
Employment, *n* (%)					<0.001
Employed	1,035 (77.0)	313 (66.6)	468 (79.9)	254 (87.9)	
Retired	25 (1.9)	5 (1.1)	15 (2.6)	5 (1.7)	
Self-employed	81 (6.0)	37 (7.9)	33 (5.6)	11 (3.8)	
Student	168 (12.5)	100 (21.3)	53 (9.0)	15 (5.2)	
Unemployed	36 (2.7)	15 (3.2)	17 (2.9)	4 (1.4)	
Employment (regrouped), *n* (%)					<0.001
Employed	1,116 (83.0)	350 (74.5)	501 (85.5)	265 (91.7)	
Student	168 (12.5)	100 (21.3)	53 (9.0)	15 (5.2)	
Unemployed or retired	61 (4.5)	20 (4.3)	32 (5.5)	9 (3.1)	
Financial comfort level, *n* (%)					0.319
Very comfortable	92 (6.8)	35 (7.4)	39 (6.7)	18 (6.2)	
Rather comfortable	523 (38.9)	184 (39.1)	234 (39.9)	105 (36.3)	
Neutral	415 (30.9)	137 (29.1)	192 (32.8)	86 (29.8)	
Rather uncomfortable	244 (18.1)	90 (19.1)	97 (16.6)	57 (19.7)	
Very uncomfortable	71 (5.3)	24 (5.1)	24 (4.1)	23 (8.0)	
Financial comfort level (binary), *n* (%)					
Rather or very uncomfortable or neutral	730 (54.3)	251 (53.4)	313 (53.4)	166 (57.4)	0.480
Healthcare provider = Yes, *n* (%)	947 (70.4)	286 (60.9)	440 (75.1)	221 (76.5)	<0.001
Religion, *n* (%)					<0.001
Agnosticism	125 (9.3)	82 (17.4)	34 (5.8)	9 (3.1)	
Atheism	137 (10.2)	75 (16.0)	38 (6.5)	24 (8.3)	
Christianity	101 (7.5)	30 (6.4)	44 (7.5)	27 (9.3)	
Islam	970 (72.1)	276 (58.7)	466 (79.5)	228 (78.9)	
Other	12 (0.9)	7 (1.5)	4 (0.7)	1 (0.3)	
Religion (regrouped), *n* (%)					<0.001
Agnosticism or other	137 (10.2)	89 (18.9)	38 (6.5)	10 (3.5)	
Atheism	137 (10.2)	75 (16.0)	38 (6.5)	24 (8.3)	
Christianity	101 (7.5)	30 (6.4)	44 (7.5)	27 (9.3)	
Islam	970 (72.1)	276 (58.7)	466 (79.5)	228 (78.9)	
Degree of religiosity, mean (SD)	2.7 (1.3)	2.4 (1.2)	2.8 (1.3)	2.8 (1.4)	<0.001

* Six respondents did not report their gender.

### 3.2 Attitudes toward posthumous organ donation

Over one-third of respondents expressed a willingness to donate their organs post-mortem, while one-fifth indicated they would refuse, and the remaining respondents left the decision to their relatives (Table 1). On average, those who would refuse or defer the decision to their family members were older than those willing to donate (40 and 38 years versus 33 years, respectively, *p* < 0.001). Compared to participants who were willing to donate, respondents who refused or left the decision to their relatives had higher proportions of those who primarily spoke Kazakh (63 and 59% vs. 33%, respectively), were married (67 and 65% vs. 48%, respectively), had children (79 and 75% vs. 48%, respectively), worked in healthcare (77 and 75% vs. 70%, respectively), and followed Islam (79 and 80% vs. 59%, respectively). In contrast, among respondents who were willing to donate, there was a higher proportion of university undergraduate and graduates (86%), students (21%), and agnostics, atheists and followers of other religions (35%, Table 1).

### 3.3 Multinomial logistic regression

The odds of leaving the decision up to relatives, compared to agreeing to donate, were significantly higher in older respondents (OR = 1.05, 95% CI: 1.03–1.06), among those who completed vocational education (OR = 1.96, 95% CI: 1.28–2.98) and high school or less (OR = 2.05, 95% CI: 1.17–3.60) and those with a higher degree of religiosity (OR = 1.20, 95% CI: 1.07–1.35, Table 2). In contrast, Russian speakers had 52% lower odds of leaving the decision to their relatives (OR = 0.48, 95% CI: 0.28–0.82). Similarly, older age (OR = 1.06, 95% CI: 1.04–1.08), lower education level (high school or less, OR = 2.91, 95% CI: 1.52–5.57; vocational education, OR = 2.54, 95% CI: 1.58–4.09), higher degree of religiosity (OR = 1.22, 95% CI: 1.07–1.40) were associated higher odds of refusing to donate. In addition, males (OR = 1.93, 95% CI: 1.31–2.84), Russians (OR = 1.80, 95% CI: 1.04–3.12) and those who felt financially “Rather or very uncomfortable or neutral” (OR = 1.44, 95% CI: 1.04–2.00) had higher odds of refusing to donate compared to agreeing to donate. On the other hand, students (OR = 0.48, 95% CI: 0.25–0.94) and Russian speakers (OR = 0.21, 95% CI: 0.08–0.53) had lower odds of refusing to donate (Table 2).

**Table 2:** Multinomial logistic regression exploring survey participant characteristics associated with attitudes toward post-mortem organ donation, Kazakhstan.

Variable	OR	95% CI	*p*-value
Leave the decision up to close relatives vs. Agree to donate			
Age	1.05	1.03–1.06	<0.001
Sex			
Female	Ref	Ref	
Male	1.30	0.94–1.80	0.122
Ethnicity			
Kazakh	Ref	Ref	
Russian	1.34	0.82–2.19	0.215
Other	1.17	0.73–1.89	0.485
Education			
High school or less	2.05	1.17–3.60	0.011
Vocational	1.96	1.28–2.98	<0.01
Bachelor’s or Master’s	Ref	Ref	
Doctoral	0.67	0.42–1.08	0.103
Employment			
Employed	Ref	Ref	
Student	0.78	0.50–1.22	0.289
Unemployed or retired	1.11	0.58–2.12	0.825
Residence			
Rural	Ref	Ref	
Urban	0.76	0.52–1.09	0.142
Spoken language			
Kazakh language	Ref	Ref	
Russian language	0.48	0.28–0.82	<0.01
Both languages or other language	1.23	0.87–1.75	0.209
Marital status			
Single			
Married	0.93	0.48–1.78	0.785
Divorced	1.00	0.60–1.66	0.905
Widowed	1.27	0.87–1.85	0.213
Degree of religiosity	1.20	1.07–1.35	<0.001
Financial comfort level			
Rather or very comfortable	Ref	Ref	
Rather or very uncomfortable or neutral	1.15	0.88–1.50	0.334
Healthcare provider			
No	Ref	Ref	
Yes	1.20	0.88–1.62	0.326
Refuse to donate vs. Agree to donate			
Age	1.06	1.04–1.08	<0.001
Sex			
Female	Ref	Ref	
Male	1.93	1.31–2.84	<0.001
Ethnicity			
Kazakh	Ref	Ref	
Russian	1.89	1.04–3.43	0.04
Other	1.80	1.04–3.12	0.04
Education			
High school or less	2.91	1.52–5.57	<0.001
Vocational	2.54	1.58–4.09	<0.001
Bachelor’s or Master’s	Ref	Ref	
Doctoral	0.86	0.49–1.51	0.625
Employment			
Employed	Ref	Ref	
Student	0.48	0.25–0.94	0.033
Unemployed or retired	0.47	0.19–1.16	0.106
Residence			
Rural	Ref	Ref	
Urban	0.92	0.60–1.42	0.725
Spoken language			
Kazakh language			
Russian language	0.21	0.08–0.53	<0.001
Both languages or other language	0.98	0.55–1.75	0.934
Marital status			
Single			
Married	0.83	0.39–1.77	0.635
Divorced	1.04	0.58–1.88	0.915
Widowed	1.20	0.76–1.88	0.426
Degree of religiosity	1.22	1.07–1.40	<0.01
Financial comfort level			
Rather or very comfortable	Ref	Ref	
Rather or very uncomfortable or neutral	1.44	1.04–2.00	0.035
Healthcare provider			
No	Ref	Ref	
Yes	1.03	0.70–1.51	0.902

### 3.4 Qualitative analysis of open-ended responses

For the qualitative analysis, after excluding irrelevant or very brief responses, a total of 954 open-ended narratives (71% of 1,345 respondents) were analyzed; no new codes appeared after the 920-th response, indicating thematic saturation. The thematic analysis identified five key themes that influence attitudes toward organ donation. Table 3 provides a summary of the recurring keywords associated with each theme.

**Table 3:** Associated keywords.

**The themes**	**Key mentioning words**
Concerns about death and afterlife	“soul,” “afterlife,” “fear,” and “God’s will.”
Uncertainty and decision-making	“not sure,” “maybe,” “need more information,” and “depends on family”
Ethical or cultural opposition	“forbidden,” “haram,” “against beliefs” and “tradition”
Trust in healthcare system	“doctors,” “corruption,” “trust issues” and “organ trafficking”
Personal choice and altruism	“my decision,” “help others,” “good deed,” and “save lives”

Concerns about death and the afterlife were frequently mentioned, with many respondents expressing fears that organ donation might interfere with their spiritual journey or contradict religious teachings. This uncertainty was compounded by a general reluctance to make a decision, with some people admitting to a lack of knowledge and preferring to leave the decision to family members. Ethical and cultural opposition also emerged as an important issue, with several respondents citing religious and traditional prohibitions as a major deterrent. Many felt that organ donation conflicted with their moral framework or societal expectations.

### 3.5 Misunderstandings and lack of knowledge

The thematic analysis of the responses revealed different motivations and concerns regarding organ donation. Among those who refused to donate for religious reasons, some respondents expressed uncertainty or lack of complete information, which led them to decide not to donate.

Lower education strongly predicted refusal (OR 2.91 for high-school-only vs. bachelor and higher) and deference (OR 2.05), mirroring many pleas for better information.

“I do not know how acceptable donation is in Islam; that’s why I answered many questions neutrally.” (Female (F), 41 years) “I cannot donate my organs, it scares me. I am very afraid that I will be held accountable for my organ donation in the afterlife, so I am against donation.” (F, 20) The level of awareness is low; I think we should start teaching about donation in schools.” (Male (M), 18)

### 3.6 Religious and ethical values

More definitive beliefs about bodily integrity after death were also observed. Islam is the majority faith (79% of those who refused vs. 59% of donors) and each one-point rise in religiosity increased refusal odds by 22% (OR 1.22, 95% CI 1.07–1.40).

“According to Islam, a deceased person should be buried with his whole body.” (F, 20) “I want to go to the other world in the same condition as I came from my mother.” (F, 41) “The organ that Allah has given me is His trust in me, and I want this organ to remain only with me.” (F, 23)

Others acknowledged the importance of organ donation for medical progress, but still refused because of religious beliefs.

“I support donation if a person has his own consent, but from a religious point of view I am against taking my body after death because I am responsible for my body in this life and I am accountable to God for my body.” (M), 28 years

### 3.7 Systemic distrust and corruption concerns

Trust in the healthcare system was also a recurring concern, with participants expressing doubts about the integrity of medical institutions and fears of organ trafficking or unethical practices.

Doubts about the transparency and ethical standards of the healthcare system were expressed by both those who gave consent and those who signed a waiver. Many respondents feared corruption, unethical practices and potential misuse of organs, which significantly influenced their decisions. Financial unease predicted lower donation willingness: respondents who felt “rather/very uncomfortable or neutral” about their finances had 44% higher odds of refusing versus agreeing to donate (OR 1.44, 95% CI 1.04–2.00).

Among those who were willing to donate, concerns about organ trafficking and unethical practices persisted, but ultimately did not affect their decision:

“I am concerned about possible corrupt practices related to donation. In particular, I am worried that my organs might be stolen and sold somewhere. But I am still willing to be a donor after my death. (M, 22) “There are many questions about the system, but that does not change the fact that it can save the lives of many people. (M, 28) “I have no trust in the health system in general and in the ethics of doctors in particular. Considering the frequent corruption scandals in health care, I think it is not safe to be a donor in Kazakhstan. At the same time, I support the idea of donation in general.”

There was also a general sense of distrust in the fairness and transparency of organ allocation, despite recognition of the value of donation:

“I understand the importance of organ donation and consider it a noble cause. However, I have questions about the level of government control over this process. Won’t each of us be targeted if we agree to donate? I would donate my organs to my children and close relatives, both during my lifetime and posthumously. But when it comes to strangers on the waiting list, how transparent is everything? Is there no manipulation of the waiting list? Can people pay bribes to get on the waiting list? Since I do not have answers to these questions, I cannot agree to organ donation. (F, 58)

“With our corrupt system, it is dangerous to develop organ donation. They can even use organs without a person’s consent. A few years ago I heard a story about ‘black’ transplant doctors.” (M, 56)

### 3.8 Supportive motivations and altruism

Conversely, some individuals framed organ donation as a deeply personal and altruistic choice, emphasizing its potential to save lives. These respondents emphasized the humanitarian and ethical responsibilities associated with donation and saw it as an opportunity to contribute to society. Exactly 470 participants (34.9%) had already registered lifetime consent; they were the youngest subgroup (mean 32.6 y) and far more often agnostic/atheist (34%) than refusers (11%).

Among those who agreed to donate, the predominant motivation was the belief that organs are no longer needed after death. Several respondents articulated this sentiment clearly.

“It will help others, if there are organs left intact, why not give them to someone who needs them. Because I will not need them after I die.” (M, 22)

“If my organs cannot serve my body, they can be useful for someone else.” (M, 30)

“Because this is my civic responsibility, and why would I need my organs after death?” (F, 24) Interestingly, some respondents were motivated by the idea that registering as an organ donor would encourage them to maintain their health and healthy lifestyle.

“I know that donating will help a lot of people and make their lives much better and longer. It also makes me take care of my health so that the recipients get healthy organs.” (M, 24) Respondents also highlighted the importance of early education and awareness campaigns to promote organ donation:

“I can save at least one person’s life. I think it’s the right thing to do! I am happy that our country is introducing such innovations and I want it to develop further. But the level of awareness is low, I think we should start from schools.” (M, 18)

Media coverage was also found to be a motivator for some individuals:

“Recently there was news in the media that donation has saved lives and improved the health of some people in the country. This news influenced my decision very much and you could even say that it inspired me.” (F, 18)

### 3.9 Family authority and decision conflict

A large group - 586 respondents (43.6%) delegated the final choice to relatives; every additional year of age raised the odds of this deference by 5% (OR 1.05). However, their motivations varied. Some respondents deferred the decision out of concern for the emotional burden their families might experience during such a difficult time:

“I would leave it to my close relatives … I care about their feelings at the time of my death.” (F, 22)

“Only loved ones care about the afterlife; I’m unlikely to care about my body.” (F, 19)

Some respondents saw this approach as a way to encourage family members to think carefully about organ donation, potentially leading to a positive decision:

“I think if I leave the choice to them, they will have a sense of responsibility and duty towards me, because I will always talk about donation and my consent during my life-time. In this way, people will believe in fair treatment of donation–not for money.” (F, 51)

Others, due to mistrust in the health care system, transferred decision-making authority to relatives and expressed concerns about prioritizing their care in critical situations:

“The decision depends on the age and the cause of death. And also there is not complete trust in doctors, there is a fear that in an emergency my life and my salvation will not be prioritized.” (M, 37)

“I would not be against donation after my death. But I do not trust our medical system. Even more, I do not trust that doctors will fight for my life.” (F, 41)

“Because they know if the doctors did all they could to save me before suggesting that my son donate my organs. And he knows that I agree with it.” (F, 43)

## 4 Discussion

The findings of this study highlight the complex interplay of cultural, religious, and systemic factors that shape public attitudes toward posthumous organ donation in Kazakhstan. By integrating both quantitative and qualitative analyses, our findings provide a multidimensional perspective on the key barriers and facilitators that influence willingness to donate.

### 4.1 Religious and cultural barriers to organ donation

A significant finding of this study was the strong negative correlation between religiosity and willingness to donate. Many respondents expressed concerns about the spiritual implications of organ donation. Similar concerns have been reported in studies conducted in other Muslim-majority countries, such as Saudi Arabia and Iran, where uncertainty about the compatibility of organ donation with Islamic teachings is a major deterrent (10, 11).

However, Islamic scholars and organizations such as the Islamic Fiqh Academy have issued fatwas supporting organ donation as an act of charity (sadaqah jariyah), suggesting that more efforts are needed to disseminate religiously informed guidance to the public.

Ethical and cultural opposition also emerged as a major barrier, with many respondents believing that organ donation goes against traditional values. This is consistent with studies from Turkey and Pakistan, where cultural identity and family influence strongly influence decisions about organ donation (12, 13). Addressing these concerns will require culturally sensitive education programs that incorporate religious perspectives and engage community leaders in discussions about the benefits of trans-plantation.

### 4.2 Trust in the healthcare system

Public distrust in the healthcare system remains a critical challenge, with a significant proportion of respondents citing concerns about organ trafficking, corruption and unethical medical practices. This finding is consistent with global trends, as distrust in medical institutions has been shown to negatively impact organ donation rates (14, 15). Countries with successful organ donation programs, such as Spain and the United Kingdom, have implemented strict legal frameworks and transparency measures to build public trust in the organ allocation system (16).

Our findings suggest that socioeconomic marginalization is closely tied to distrust of institutions. Participants from lower-education and lower-income backgrounds more frequently referenced corruption, unfair distribution, and fear of exploitation. While many expressed theoretical agreements with organ donation, they rejected the idea in practice due to a lack of trust. These results suggest that public support for organ donation may depend not only on knowledge or religion, but also on perceptions of fairness and equity in health governance.

Kazakhstan could adopt similar strategies by establishing independent oversight committees, increasing transparency in organ procurement, and ensuring that public trust is strengthened through clear communication and ethical medical practices.

### 4.3 Attitudes among medical professionals and students

Healthcare professionals and students play a crucial role in shaping public perceptions of organ donation. While healthcare professionals were slightly more willing to donate than the general population, the difference was not as pronounced as might be expected. Like the general public, many health professionals cited religious or ethical concerns and mistrust of the health care system as reasons for refusing or hesitating to provide lifetime consent. In addition, a significant proportion of health professionals opted to leave the decision to family members, mirroring the approach of the general population.

This finding is consistent with research from other countries where health professionals, despite their medical knowledge, do not always act as proactive advocates for organ donation. Studies from Turkey and India have found that even among healthcare professionals, concerns about ethical issues, family influence, and systemic mistrust can limit personal commitment to donation (17–19). These findings underscore the need for targeted awareness programs within the medical community to ensure that healthcare professionals are not only informed, but also encouraged to take an active role in promoting a culture of donation.

Other recent studies have shown that medical students and professionals are more likely to support organ donation due to their familiarity with the transplantation process and its benefits (20). However, training programs that equip healthcare professionals with effective communication strategies could enhance their role as advocates for organ donation.

Medical students, in particular, are an important target group for educational interventions. Studies from India and Egypt suggest that integrating organ donation awareness into medical curricula can significantly improve knowledge and willingness to donate (21, 22). Kazakhstan’s medical institutions could benefit from incorporating similar training modules to ensure that future healthcare providers are well equipped to address public concerns and dispel myths surrounding organ transplantation.

### 4.4 The role of media and health communication

The perspectives gathered in the thematic analysis underscore the need for targeted educational initiatives, transparency in medical processes, and culturally sensitive out-reach to address misconceptions and build public trust in organ donation programs.

Media portrayals play a critical role in shaping public perceptions of organ donation. In our study, respondents frequently mentioned concerns influenced by misinformation, which is consistent with existing literature showing that media exposure can either facilitate or hinder public acceptance of organ donation (23). Research has shown that negative portrayals of organ procurement, particularly in film and television, contribute to mistrust and fear (24). In contrast, well-structured media campaigns have successfully increased organ donor registration in countries such as the United States and South Korea (25, 26).

Although the world’s religions generally support organ donation for a variety of reasons, religious beliefs can be a serious barrier to making a final decision (27). This has more to do with doubt and lack of clear knowledge than outright opposition. To address these uncertainties, religious scholars and medical professionals must work together to disseminate accurate information about the ethical and medical aspects of organ donation (13).

Healthcare organizations must proactively manage their media relations to maintain public trust. Regular communication with journalists, media training for transplant professionals, and strategic crisis management planning can prevent misinformation from escalating into public mistrust (15). For example, the National Transplant Organization (ONT) in Spain has successfully implemented rapid response strategies to counter media scandals and ensure that public confidence in organ transplantation remains stable (28). Kazakhstan and other countries could benefit from adopting similar strategies by promoting transparency and ensuring that media narratives about organ donation emphasize its societal benefits rather than isolated negative cases.

Given the positive correlation between being a health professional and willingness to donate, health professionals can serve as influential advocates in raising awareness and fostering trust in the donation process (29). When healthcare professionals engage with the media, whether through interviews, educational campaigns, or social media content, they can help counter misinformation and emphasize the ethical and life-saving aspects of transplantation.

In addition, social media, which is widely used not only by young people but also by older generations, can serve as a powerful tool for spreading awareness and fostering discussion on the issue. Social media campaigns featuring testimonials from transplant recipients, religious endorsements and insights from medical experts could help dispel misconceptions and promote informed decision-making. Using digital platforms for interactive Q&A sessions with religious leaders and transplant surgeons can further bridge the gap between medical knowledge and spiritual concerns, ultimately increasing public acceptance of organ donation and promote informed decision-making.

An important consideration that emerged from the study is the role of language in organ donation attitudes. A notable trend was observed in the responses–Kazakh-speaking participants were more likely to express hesitation or opposition to organ donation compared to Russian-speaking participants. This may be due to the fact that Kazakh language media is still developing, leading to a potential gap in access to reliable and engaging information on medical topics, including organ donation.

The importance of communicating health information in one’s native language or mother tongue has been well documented in global health communication research (30, 31). Studies have shown that people are more likely to engage with, trust, and act on information when it is presented in their primary language (32). The lack of organ donation awareness campaigns in Kazakh may contribute to misunderstanding, reluctance, and reliance on cultural narratives rather than evidence-based medical information. To address this gap, Kazakhstan should prioritize the expansion of Kazakh language health communication initiatives related to organ donation. By integrating culturally and linguistically tailored messages into public health communications, Kazakhstan can bridge the information gap and potentially increase acceptance of organ donation among Kazakh-speaking communities.

### 4.5 Policy implications and recommendations

The results of this study highlight several critical areas where targeted interventions could improve public perceptions and participation in organ donation. Addressing religious concerns, building trust in the healthcare system, improving medical education, using the media to raise public awareness, and implementing policy reforms are key areas for action. A coordinated effort in these areas will be essential to create a more supportive environment for organ donation in Kazakhstan.

Working with religious scholars and disseminating official fatwas supporting organ donation can help address theological concerns and provide clarity to potential donors.

Implementing transparent organ allocation policies, establishing independent over-sight bodies, and ensuring ethical medical practices will be essential to addressing public distrust.

Training programs for healthcare professionals and students should emphasize the importance of organ donation advocacy and equip them with the skills to communicate effectively with patients and the public.

The use of media psychology strategies such as positive storytelling, myth-busting initiatives and educational documentaries can help change public perceptions and counteract misinformation.

Reviewing and refining Kazakhstan’s organ donation policies, including opt-in versus opt-out systems, can provide a structural framework that supports increased donor registration.

## 5 Limitations of the study

Although qualitative survey data is generally less detailed than interview data, our anonymous online format allowed a diverse group of participants to share their opinions on a sensitive topic. This method allowed us to collect over 900 open-text responses, a rarity in organ donation literature. While limited in depth, this breadth adds valuable insights into population-level attitudes. However, several limitations should be considered.

First, the study relied on a convenience sampling method, which may not be fully representative of the entire population. The use of convenience sampling may limit the generalizability of our findings. Individuals with prior interest in health-related topics or organ donation may have been more likely to participate, introducing potential selection bias. Urban residents and individuals with higher levels of education were more likely to participate, potentially biasing the results. A more diverse and random sample would provide a more balanced perspective. Second, responses were based on self-reported data, which can be influenced by social desirability bias. Participants may have expressed more favorable attitudes toward organ donation than they actually hold in practice. Future studies should include behavioral measures to assess actual donation intentions and explore stratified or randomized sampling approaches.

Another limitation is the focus on Islamic beliefs, as Kazakhstan is a predominantly Muslim country. While religious concerns were explored in depth, the perspectives of minority religious groups were not fully explored. Including a broader religious representation in future research could provide a more nuanced understanding of faith-based attitudes toward organ donation.

In addition, the study was cross-sectional, measuring attitudes at a single point in time. This limits our ability to assess how public perceptions evolve, particularly in response to awareness campaigns or policy changes. A longitudinal approach would help track changes in attitudes and identify the impact of interventions over time.

Finally, while qualitative analysis enriched our understanding of public concerns, open-ended responses varied in depth and clarity. Some participants may have interpreted organ donation terminology differently, leading to inconsistencies in the thematic analysis. More structured qualitative interviews could provide deeper insights into personal motivations and barriers.

Despite these limitations, this study lays a strong foundation for future research and policy development. Expanding the scope of participants, incorporating longitudinal methods, and exploring behavioral aspects of organ donation could further enhance our understanding and contribute to effective strategies for increasing donor registration in Kazakhstan.

## 6 Conclusion

This study provides valuable insights into the complex factors influencing attitudes towards organ donation in Kazakhstan. By comparing our findings with international research, we highlight key barriers such as religious concerns, mistrust of health care, and cultural opposition, and identify potential solutions through education, policy change, and media engagement. Addressing these challenges through evidence-based interventions will be critical to improving public acceptance of organ donation and ultimately increasing donor registration rates. Future research should explore long-term strategies for implementing these recommendations and evaluating their effectiveness in the context of Kazakhstan.

## Data Availability

The raw data supporting the conclusions of this article will be made available by the authors, without undue reservation.
